# Protocol to visualize and quantify the COPII concentration and anterograde transport of nascent G protein-coupled receptors

**DOI:** 10.1016/j.xpro.2024.102955

**Published:** 2024-03-13

**Authors:** Xin Xu, Guangyu Wu

**Affiliations:** 1 Department of Pharmacology and Toxicology, Medical College of Georgia, Augusta University, Augusta, GA 30912, USA

**Keywords:** Cell Biology, Cell-based Assays, Molecular Biology

## Abstract

Here, we present a protocol for visualization and quantification of the recruitment of newly synthesized G protein-coupled receptors (GPCRs) to coat protein complex II vesicles and GPCR transport from the endoplasmic reticulum through the Golgi to the cell surface in the retention using the selective hooks assay. We describe steps for plasmid construction, cell transfection, transport synchronization, confocal microscope imaging, and quantification. This protocol is also applicable for studying the transport of non-GPCR cargoes.

For complete details on the use and execution of this protocol, please refer to Xu et al.^1^^,^^2^

## Highlights

• Application of the RUSH system in studying the forward delivery of nascent GPCRs
• Steps to visualize the concentration of GPCRs in COPII vesicles
• Introductions for characterizing the ER-to-Golgi transport of GPCRs
• Guidance on measuring the post-Golgi traffic of GPCRs

## Abstract (editor highlights)

Publisher’s note: Undertaking any experimental protocol requires adherence to local institutional guidelines for laboratory safety and ethics.

## Graphical abstract

**Figure:**
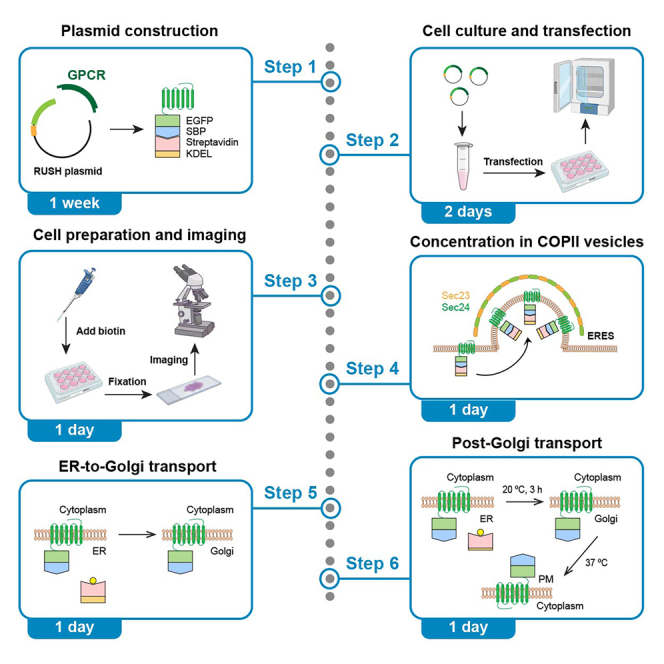

## Before you begin

Coat protein complex II (COPII) vesicles are well characterized to exclusively mediate the export of newly synthesized proteins from the endoplasmic reticulum (ER).^3^ G protein-coupled receptors (GPCRs) constitute the largest superfamily of cell surface signaling proteins and are important targets of the drugs on the market.^4^^,^^5^ However, it remains largely unknown how GPCRs are recruited to COPII vesicles from the ER where they are born, and then anterogradely transported to the cell surface, the functional destination for most GPCRs, *en route* pass through the Golgi apparatus where they may undergo post-translational modifications.^6^^,^^7^ When compared with non-GPCR plasma membrane proteins and secretory cargoes, GPCRs may use specific motifs and directly interact with the components of transport machineries and regulatory proteins to control their forward delivery.^8^^,^^9^^,^^10^^,^^11^^,^^12^^,^^13^^,^^14^^,^^15^^,^^16^^,^^17^

**Figure 1:**
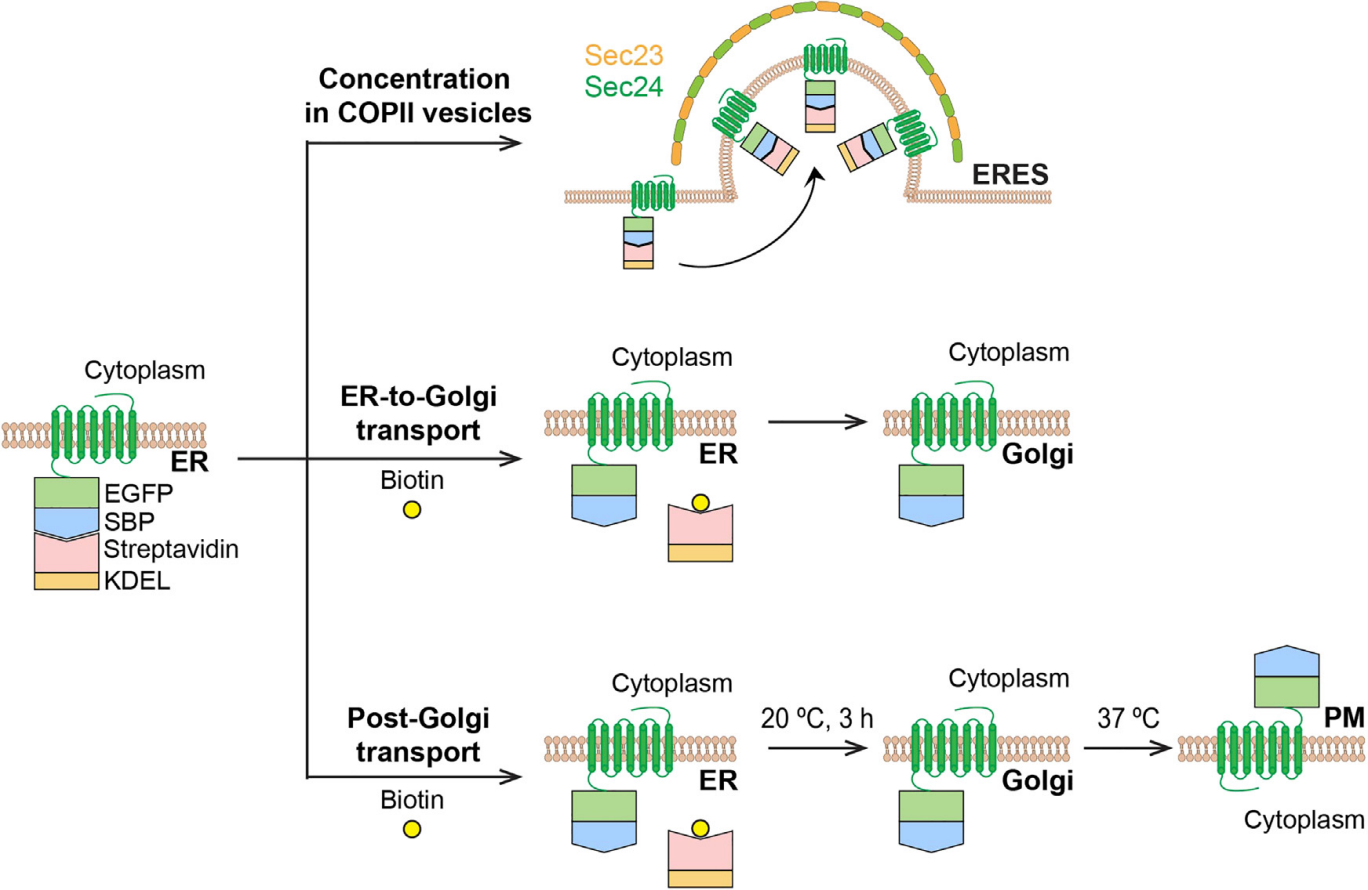
Schematic diagram to study GPCR transport using the RUSH system

Although a number of methods, such as radioligand binding and pulse chase,^18^^,^^19^^,^^20^ have been employed to study the regulation of GPCR biosynthesis in cell, recently established retention using the selective hooks (RUSH) assays can be used to visualize GPCR capture by COPII vesicles and study their transport dynamics between intracellular compartments.^1^^,^^2^^,^^21^^,^^22^ In this protocol, we describe steps to detect the recruitment of nascent GPCRs to COPII vesicles and their anterograde transport in RUSH assays.^21^ In the RUSH system, GPCR of interest is tagged with a streptavidin binding peptide (SBP) at the N-terminus and with a fluorescent protein at either terminus and the KDEL sequence used as a hook for retention in the ER is conjugated with streptavidin (Figure 1). After RUSH plasmids are delivered into cells and the receptors are synthesized in the ER, the interaction between SBP and streptavidin makes the nascent receptors retained in the ER. Once biotin is added to disrupt the interaction, the receptors can leave the ER and move forward to the Golgi (Figure 1).^2^^,^^21^^,^^23^

In our recent studies, we have generated a series of GPCRs in RUSH plasmids^1^^,^^2^^,^^22^ and found that, although all GPCRs studied are unable to leave the ER in RUSH assays in the absence of biotin, only a few GPCRs are recruited to and highly concentrated at the ER exist sites (ERES) on the ER membrane from where COPII vesicles are budded.^1^ We have analyzed the ER-to-Golgi transport kinetics of GPCRs in cells after incubation with biotin.^1^^,^^2^^,^^22^ In addition, we have studied the post-Golgi transport of GPCRs using the RUSH system in combination with temperature-induced transport control (Figure 1).^2^

### Preparation of solutions and cell culture medium

**Timing: 6 h**

1. 0.15 M NaCl: Dissolve 1.35 g of NaCl and make the final volume to 50 mL. Filter the solution with 0.22 μm filter and store at 4°C.
2. 400 mg/mL CHX: Dissolve 83 mg of CHX into 207 μL of DMSO. Aliquot and store at −20°C.
3. 7.5 mM PEI:
  a. Add 54 mg of PEI and 1 mg of phenol red to 80 mL of sterile water.
  b. Adjust pH to 2.0 with HCl. Stir until all particles are dissolved.
  c. Add 5 mL of 1 M HEPES and adjust pH to 7.0 with 1 M NaOH.
  d. Add sterile water to make the final volume to 100 mL.
  e. Filter the solution with 0.22 μm filter.
  f. Aliquot and store at 4°C or −20°C for long term use.
4. 4 mM biotin:
  a. Add 50 mg of biotin into 45 mL of sterile water.
  b. Shake until biotin is completely dissolved.
  c. Make the final volume to 50 mL. Filter with 0.22 μm filter.
  d. Aliquot and store at 4°C or −20°C for long term use.
5. 4% PFA:
  a. Add 4 g of PFA to 90 mL of PBS in a glass bottle with lid.
  b. Put the bottle at 60°C in water bath until PFA is dissolved.
  c. Make the final volume to 100 mL.
  d. Filter the solution with filter paper. Aliquot and store at −20°C.
6. DMEM containing 10% FBS and 1% P/S: Add 50 mL of FBS and 5 mL of P/S to 445 mL of DMEM. Mix well and store at 4°C. Pre-warm the medium at 37°C in water bath before use.
7. DMEM containing 10% FBS, 40 μM biotin and 400 μg/mL CHX: dilute FBS at 1:10, 4 mM biotin solution at 1:100 and 400 mg/mL CHX solution at 1:1000 with DMEM. For example, to make the final volume of 10 mL, add 1 mL of FBS, 100 μL of biotin and 10 μL of CHX to 8.89 mL of DMEM.
8. DMEM containing 10% FBS and 400 μg/mL CHX: dilute FBS at 1:10 and 400 mg/mL CHX solution at 1:1000 with DMEM. For example, to make the final volume of 10 mL, add 1 mL of FBS and 10 μL of CHX to 8.99 mL of DMEM.

## Key resources table

**Table:** 

REAGENT or RESOURCE	SOURCE	IDENTIFIER
**Chemicals, peptides, and recombinant proteins**		

Ampicillin sodium salt	Sigma-Aldrich	Cat# A0166
Cycloheximide (CHX)	Thermo Fisher Scientific	Cat# AAJ6690103
D-biotin	Thermo Fisher Scientific	Cat# AC230090010
DH5α competent cells	Thermo Fisher Scientific	Cat# 18265017
Dimethyl sulfoxide (DMSO)	Sigma-Aldrich	Cat# MX1458
Dulbecco’s modified Eagle’s medium (DMEM)	HyClone	Cat# SH30243.01HI
Fetal bovine serum (FBS)	HyClone	Cat# SH30396.03HI
FseI	New England Biolabs	Cat# R0588S
HCl	Sigma-Aldrich	Cat# 320331
HEPES (1 M)	Gibco	Cat# 15630056
NaCl	Thermo Fisher Scientific	Cat# BP358
NaOH (1 M)	Sigma-Aldrich	Cat# 1091371003
Paraformaldehyde (PFA)	J.T.Baker	Cat# 02-003-575
Penicillin-streptomycin solution (P/S)	HyClone	Cat# SV30010
Phenol red	Sigma-Aldrich	Cat# 114529
Phosphate-buffered saline (PBS, 10×)	Thermo Fisher Scientific	Cat# BP3994
Platinum Taq DNA polymerase	Thermo Fisher Scientific	Cat# 10966018
Polyethylenimine (PEI)	Polysciences	Cat# 23966
Poly-L-lysine solution	Sigma-Aldrich	Cat# P4832
ProLong gold antifade mountant	Thermo Fisher Scientific	Cat# P36930
ProLong gold antifade mountant with DNA stain DAPI	Thermo Fisher Scientific	Cat# P36931
QIAprep spin miniprep kit	QIAGEN	Cat# 27106
QIAquick gel extraction kit	QIAGEN	Cat# 28706
T4 DNA ligase (1 U/μL)	Thermo Fisher Scientific	Cat# 15224017
XbaI	New England Biolabs	Cat# R0145S

**Experimental models: Cell lines**		

HeLa	ATCC	Cat# CRM-CCL-2
Human embryonic kidney (HEK) 293	ATCC	Cat# CRL-1573

**Recombinant DNA**		

α2A-AR	Xu et al.^1^	N/A
AT2R	Xu et al.^1^	N/A
DsRed2-ER	Duvernay et al.^24^	N/A
DsRed-Sec24D	Xu et al.; Dong et al.^1^^,^^20^	N/A
PmTurquoise2-Golgi	Addgene	Cat# 36205
Str-KDEL_SBP-EGFP-Ecadherin	Addgene	Cat# 65286

**Software and algorithms**		

Excel	Microsoft	https://www.microsoft.com
Fiji	NIH	https://imagej.net/software/fiji/
Las X 4.3.0	Leica Microsystems	https://www.leica-microsystems.com/

**Other**		

0.22 μm syringe-driven filter unit	Millipore	Cat# SLMP025SS
12-well cell culture plates	Thermo Fisher Scientific	Cat# 130185
Inkjet microscope slides	Thermo Fisher Scientific	Cat# 12550100
Leica Stellaris 5 confocal microscope	Leica Microsystems	N/A
Microscope coverslips (18 mm)	Thermo Fisher Scientific	Cat# 12545100

## Step-by-step method details

### Construction of RUSH plasmids of GPCRs

**Timing: 1 week**

This part describes the generation of RUSH plasmids of GPCRs by using angiotensin II type 2 receptor (AT2R) as an example. cDNA coding AT2R is amplified by PCR, digested with Fsel and Xbal enzymes and ligated to the backbone of the RUSH plasmid Str-KDEL_SBP-EGFP-Ecadherin after digestion with the same enzymes to release E-cadherin.

1. Primers for amplifying human AT2R are as below:

Forward: 5′-GATCGGCCGGCCAATGAAGGGCAACTCC-3’.

Reverse: 5′-GATCTCTAGATTAAGACACAAAGGTCTCCATTTCTC-3’.

All primers were synthesized by Integrated DNA Technologies.

2. Conduct PCR as follows by using Platinum Taq DNA polymerase kit.

**Table:** 

Reagent	Final concentration	Amount
10× PCR buffer	1×	5 μL
50 mM MgCl_2_	1.5 mM	1.5 μL
10 mM dNTP mix	0.1 mM	0.5 μL
10 μM forward primer	0.2 μM	1 μL
10 μM reverse primer	0.2 μM	1 μL
AT2R	200 ng/reaction	1 μL
Platinum Taq DNA Polymerase	2 U/reaction	0.2 μL
Sterile H_2_O	N/A	39.8 μL
Total	N/A	50 μL

3. Set up PCR program and run.

**Table:** 

Steps	Temperature	Time	Cycles
Initial denaturation	94°C	3 min	1
Denaturation	94°C	30 s	35
Annealing	60°C	30 s	
Extension	72°C	1.1 min	
Final extension	72°C	10 min	1
Hold	4°C	indefinite

4. Separate the PCR product (1,115 bp) by 1% agarose gel electrophoresis.
  a. Extract DNA by using the QIAquick gel extraction kit.
  b. Elute DNA with 50 μL of elution buffer.
  c. Measure the DNA concentration.
5. Digest the PCR product and the plasmid Str-KDEL_SBP-EGFP-Ecadherin for 30 min at 37°C as follows.

**Table:** 

Reagent	Final concentration	Amount
10× rCutSmart buffer	1×	2.5 μL
PCR product or Str-KDEL_SBP-EGFP-Ecadherin	500 ng/reaction	10 μL
Fsel	2 U/reaction	1 μL
Xbal	2 U/reaction	1 μL
Sterile H_2_O	N/A	10.5 μL
Total	N/A	25 μL

6. Isolate digested PCR product (1,104 bp) and Str-KDEL_SBP-EGFP (5,924 bp) following step 4.
7. Conduct ligation reaction for 1 h as follows.

**Table:** 

Reagent	Final concentration	Amount
5× DNA ligase reaction buffer	1×	2 μL
Digested PCR product	60 ng/reaction	1.5 μL
Str-KDEL_SBP-EGFP	100 ng/reaction	1 μL
T4 ligase	0.2 U/reaction	0.2 μL
Sterile H_2_O	N/A	5.3 μL
Total	N/A	10 μL

***Note:*** Use the molar ratio of the PCR product:Str-KDEL_SBP-EGFP at about 3:1 in ligation reaction.

8. Transform 25 μL of subcloning efficiency DH5α competent cells with 1.5 μL of ligation solution according to the manufacturer procedures (https://www.thermofisher.com/order/catalog/product/18265017?SID=srch-srp-18265017). Spread the transformation medium on LB plate containing 100 μg/mL ampicillin and incubate the plate at 37°C for 12–16 h.
9. Pick several single colonies (usually 3) each into 3 mL of LB medium containing 100 μg/mL ampicillin and grow them for 16–20 h at 37°C with constant shaking at 250 rpm.
10. Purify plasmids using the QIAprep spin miniprep kit, measure plasmid concentration and verify plasmids by nucleotide sequence analysis (Azenta Life Sciences).

### Cell transfection

**Timing: 2 days**

This part describes the procedure of cell transfection by using PEI solution. In our studies, both HEK293 and HeLa cells are used.

11. Put one coverslip (with 18 mm diameter) in each well of 12-well plates and add 2 mL of poly-L-lysine solution. After incubation at 37°C for 15 min, remove poly-L-lysine solution. Wash coverslips with sterile water twice and let them dry.
12. Seed the cells onto 12-well plates with coated coverslips at about 30% confluence. Grow the cells in 1 mL of DMEM containing FBS and P/S for 16 h at 37°C, 5% CO_2_.

***Note:*** About 5 × 10^5^ HEK293 cells and 2 × 10^5^ HeLa cells are needed for each well.

13. Transfect the cells by using PEI solution. The total amount of plasmids used for each well is 500 ng (with a volume of 0.5–2 μL).
  a. For each transfection, add plasmids into 21 μL of 0.15 M NaCI solution in one Eppendorf tube for 5 min.
  b. In another tube, add 4 μL of 7.5 mM PEI into 17 μL of 0.15 M NaCI solution for 5 min.
  c. Combine two solutions and incubate the mixture for 15 min.
  d. Distribute all mixture into the cell culture medium.
14. Incubate the cells for 6–8 h at 37°C, 5% CO_2_.
15. Aspirate the medium, add 1 mL of fresh DMEM containing FBS and P/S and culture the cells for additional 20 h at 37°C, 5% CO_2_.

### Characterization of RUSH plasmids of GPCRs

**Timing: 3 days**

In this part, the cells are transfected with the RUSH plasmid Str-KDEL_SBP-EGFP-GPCR together with ER or Golgi marker, and then treated with biotin to initiate the transport from the ER and with CHX to block the synthesis of new receptors. After cell fixation, ER expression of the receptors via the RUSH system in the absence of biotin is indicated by their colocalization with the ER marker, whereas the receptor transport to the Golgi after biotin induction is determined by receptor colocalization with the Golgi marker.

16. To study GPCR colocalization with the ER marker, transfect the cells with 250 ng of Str-KDEL_SBP-EGFP-GPCR together with 250 ng of an ER marker (such as DsRed2-ER) for 20 h by using PEI solution following steps 11–15. Remove the medium and wash the cells twice with 1 mL of cold PBS.
17. To fix the cells, add 1 mL of 4% PFA solution for 15 min. Aspirate the PFA solution and wash the cells three times each with 1 mL of cold PBS for 5 min with gentle shaking.
18. Add one drop of ProLong Gold Antifade Mountant with DAPI on InkJet microscope slides.
  a. Pick up the coverslips from each well with tweezers or something else and put the side with the cells on the mountant.
  b. Remove the extra mountant with paper.
  c. Put the slides in a cardboard slide tray and keep the slides at 4°C.
19. To study GPCR colocalization with the Golgi marker after biotin induction, the cells are transfected with 250 ng of Str-KDEL_SBP-EGFP-GPCR together with 250 ng of a Golgi marker (such as pmTurquoise2-Golgi) by using PEI solution for 20 h following step 11–15.
20. Remove the medium and add 1 mL of DMEM containing FBS, biotin and CHX to induce receptor export from the ER for 30 min

***Note:*** CHX is not essential at this step, but it is very important when studying the ER-Golgi-surface transport after biotin induction. As such, it is suggested to use CHX in all experiments.

21. Aspirate the medium and wash the cells with cold PBS. Fix the cells and mount the coverslips following steps 17–18.

***Note:*** If pmTurquoise2-Golgi is used as a Golgi marker, mount the slides using ProLong gold antifade mountant without DAPI.

22. Image acquisition using the Leica Stellaris 5 confocal microscope with LAS X software.
  a. Set the excitation laser power at 405 nm for DAPI or pmTurquoise2, 488 nm for GFP and 561 nm for DsRed.
  b. Focus the 63× oil objective on cells expressing GFP-tagged receptors.
  c. Adjust the gain to 20%–30%.
  d. Capture pictures using frame 1024 × 1024 and speed 600.

**Figure 2:**
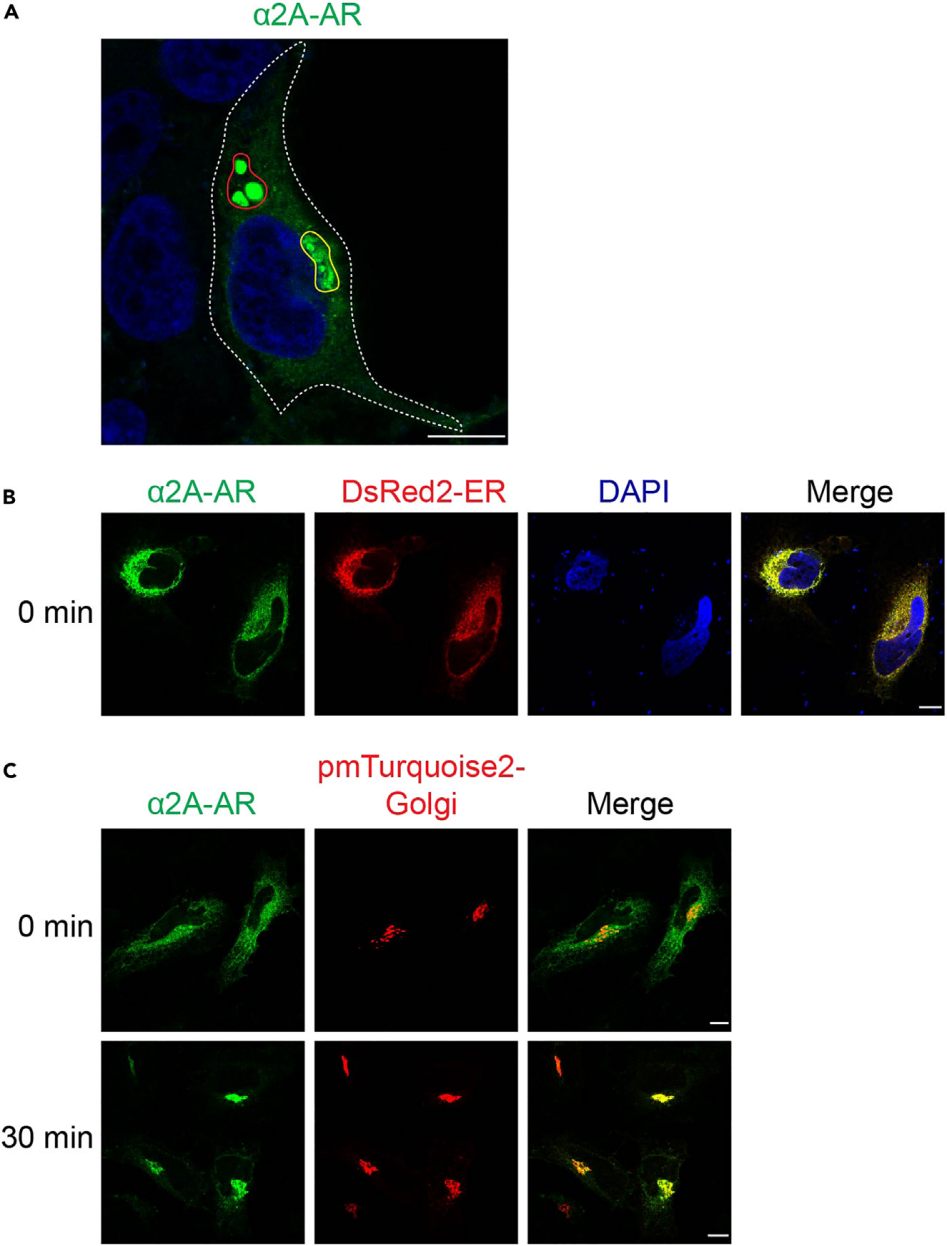
Characterization of RUSH plasmids in GPCR export from the ER (A) Possible aggregation of GPCRs in cell. HeLa cells were transfected with Str-KDEL_SBP-EGFP-α_2A_-AR and treated with biotin for 30 min. The area containing the receptors that are transported to the Golgi is denoted by the yellow line, whereas the area containing the receptors that likely form aggregates is denoted by the red line. (B) Colocalization of α_2A_-AR with the ER marker DsRed2-ER before biotin induction in HeLa cells. (C) Colocalization of α_2A_-AR with the Golgi marker pmTurquoise2-Golgi before (0 min) and after biotin treatment for 30 min in HeLa cells. Scale bars, 10 μm.

**CRITICAL:** The cells with receptor aggregation should not be chosen for imaging. It is very important to distinguish the receptors that are transported to the Golgi from those that form aggregates (Figure 2A).

**CRITICAL:** The cells that express the receptors at extremely high levels should also be excluded.

***Note:*** At least 10 pictures should be taken from each coverslip.

23. Export images acquired using LAS X software with 10 μm scale bar as TIFF files.

***Note:*** α_2A_-Adrenergic receptor (α_2A_-AR) is used as an example to analyze the colocalization of GPCRs with the ER and the Golgi markers before and after biotin addition. Before biotin addition (0 min), α_2A_-AR is colocalized with DsRed2-ER, demonstrating that α_2A_-AR is hooked in the ER in the RUSH system in the absence of biotin (Figure 2B). After biotin addition for 30 min, α_2A_-AR is mainly colocalized with pmTurquoise2-Golgi, indicating that α_2A_-AR can normally transport from the ER to the Golgi in the RUSH system in the presence of biotin (Figure 2C).

### Visualization of the concentration of GPCRs in COPII vesicles

**Timing: 4 days**

In this part, the cells are transfected with GPCR RUSH plasmids together with the COPII marker Sec24D. As compared with HEK293 cells, HeLa cells have relatively large cytoplasm and flat, thus they are better cell models for experiments to visualize COPII vesicles.

**Figure 3:**
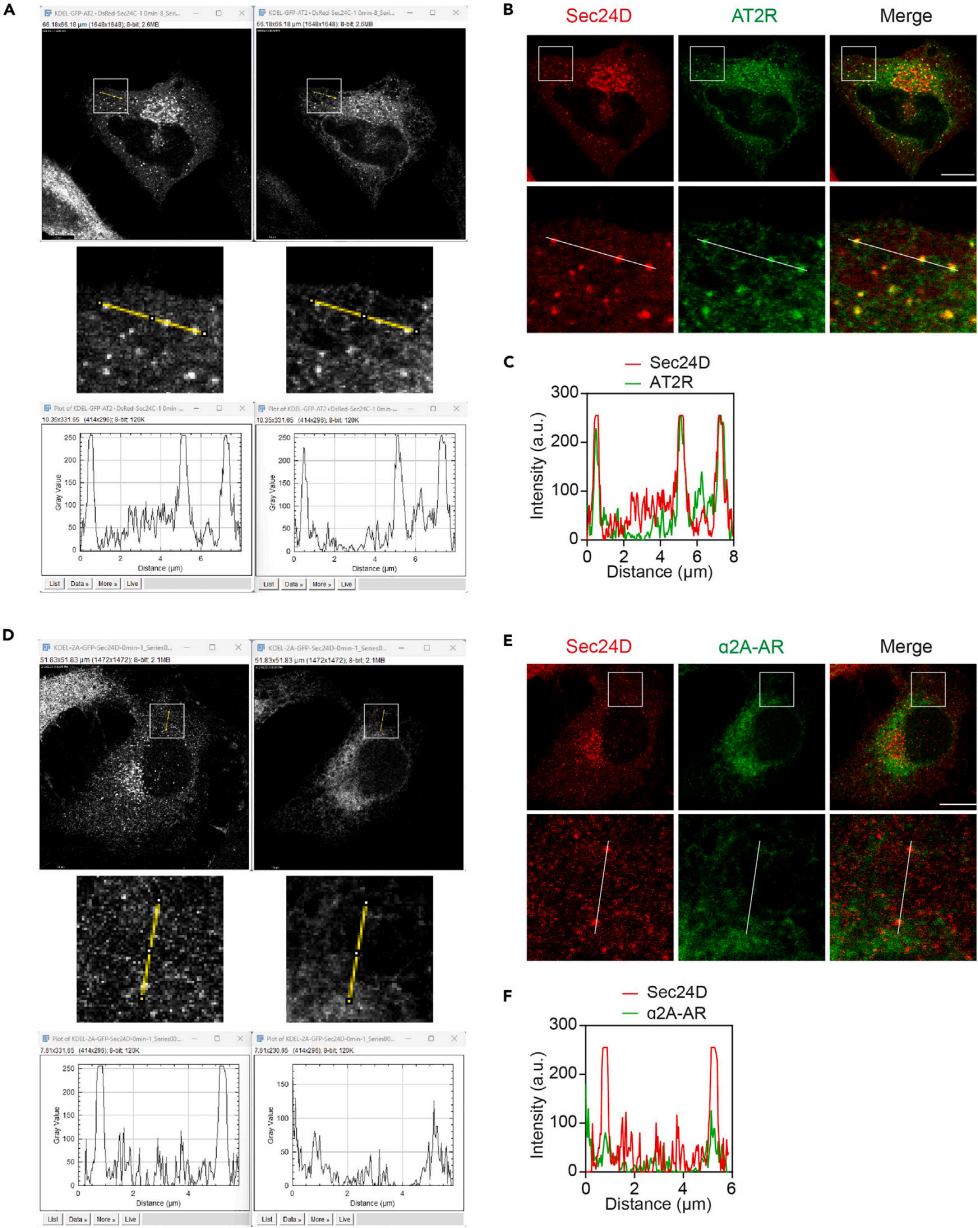
Analysis of GPCR recruitment to COPII vesicles (A) Identification of COPII vesicles and measurement of the intensities of Sec24D and AT2R in HeLa cells. (B) Colocalization of Sec24D and AT2R at the ERES. (C) Profile plot of the ROI in (B). (D) Identification of COPII vesicles and measurement of the intensities of Sec24D and α_2A_-AR in HeLa cells. (E) Colocalization of Sec24D and α_2A_-AR at the ERES. (F) Profile plot of the ROI in (E). The cells were transfected with Str-KDEL_SBP-EGFP-AT2R (A–C) or Str-KDEL_SBP-EGFP-α_2A_-AR (D–F) together with DsRed-Sec24 for 20 h before cell fixation and imaging. Scale bars, 10 μm.
  h. In the GPCR channel, click the saved ROI and plot profile to obtain the profile of GPCR intensity (Figures 3A and 3D).
  i. Copy all data and save them in Excel.

***Note:*** The scale should be re-set in each image when using Fiji.

24. Transfect the cells with 250 ng of Str-KDEL_SBP-EGFP-GPCR plus 250 ng of DsRed-Sec24D following steps 11–15.
25. After 20 h transfection, remove the medium and wash the cells twice with 1 mL of cold PBS. Fix the cells and mount the coverslips following steps 17–18.
26. Image acquisition following steps 22–23.
27. Analyze GPCR recruitment to COPII vesicles using Fiji software.
  a. Open merged images.
  b. Draw a line along the scale bar.
  c. Click set scale and change the number in know distance to 10 and the unit of length to μm. The frame of this image should be then changed to μm × μm.
  d. Split the channels and the images will then turn to black and white.
  e. Draw a line crossing two or three puncta in the picture of Sec24D channel.
  f. Add this line in the ROI manager for analyzing the same position at different channels.
  g. Click plot profile to get the profile of Sec24D intensity (Figures 3A and 3D).

28. Data presentation. AT2R and α_2A_-AR are used here as examples to analyze the colocalization of GPCRs with Sec24D. AT2R is strongly colocalized with Sec24D (Figures 3B and 3C), whereas localization of α_2A_-AR and Sec24D are apparently not overlaid (Figures 3E and 3F). These data demonstrate that AT2R, but not α_2A_-AR, is concentrated in COPII vesicles.

### Measuring the ER-to-Golgi transport of GPCRs

**Timing: 4 days**

To study the ER-to-Golgi transport of GPCRs, the cells are transfected with RUSH plasmids of GPCRs and treated with biotin for different periods of time (Figure 1). As individual GPCRs studied here are characterized by using the ER and Golgi markers as described in the section “characterization of RUSH plasmids of GPCRs”, the coexpression together with Golgi markers may not be necessary.

**Figure 4:**
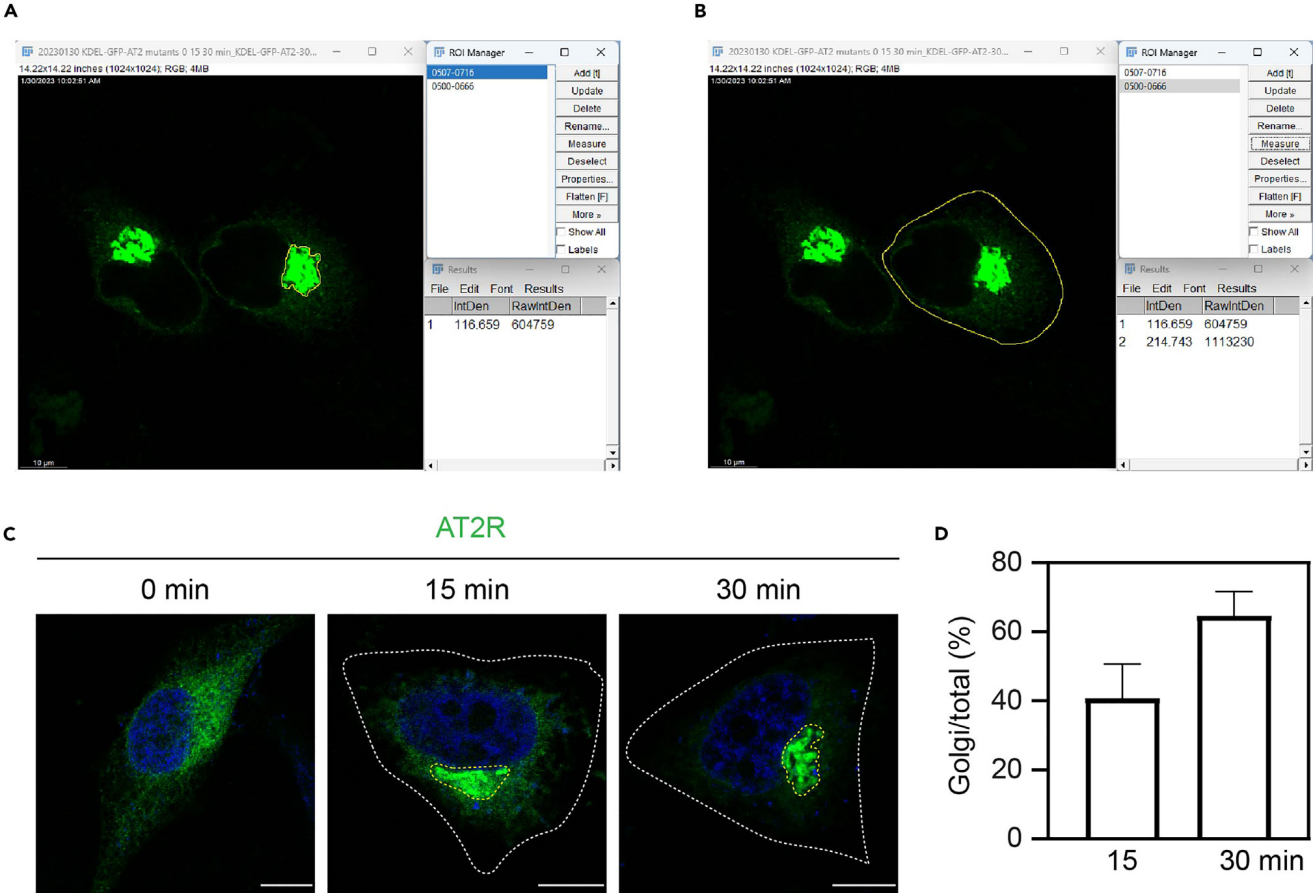
Analysis of the ER-to-Golgi transport of AT2R (A) Measurement of AT2R expression at the Golgi. (B) Measurement of AT2R expression in the whole cell. (C) ER-to-Golgi transport of AT2R in HeLa cells. The cells were transfected with Str-KDEL_SBP-EGFP-AT2R for 20 h and then treated with biotin for 15 and 30 min. (D) Quantification of the Golgi/total expression ratio of AT2R at 15 and 30 min after biotin incubation. The quantitative data are expressed as mean ± SD (n = 30-37 cells from 3 experiments). Scale bars, 10 μm.
  f. Circle the whole cell with freehand selections and measure the RawIntDen as the whole cell expression of the receptor (Figure 4B).
  g. Calculate the Golgi/total expression ratios of the receptor.

***Note:*** AT2R is used as an example to analyze the ER-to-Golgi transport (Figure 4). The receptors that are highly concentrated in the perinuclear regions are considered to be transported to the Golgi (Figure 4A). The Golgi/total expression ratio of AT2R at 15 min after biotin incubation is about 40% and this ratio increases to 60%–70% after 30 min induction (Figures 4C and 4D).

29. Transfect the cells with 500 ng of Str-KDEL_SBP-EGFP-GPCR by using PEI solution following steps 11–15.
30. After 20 h transfection, remove the medium and add 1 mL of DMEM containing 10% FBS, 40 μM biotin and 400 μg/mL CHX. To study the transport kinetics, the cells are treated for different time periods (for example, 10, 20, 30 and 40 min).
31. Remove the medium and wash the cells twice with 1 mL of cold PBS. Fix the cells and mount the coverslips following steps 17–18.
32. Image acquisition following steps 22–23.
33. Measure receptor expression in the Golgi and the whole cell.
  a. Adjust the intensity of receptor channel until the background can be seen.
  b. Export images in LAS X with receptor channel only.
  c. Open the exported images in Fiji software.
  d. Use the freehand selections to circle the area with concentrated receptors and add this area to ROI manager.
  e. Click measure to get the RawIntDen as the Golgi expression of the receptor (Figure 4A).

### Measuring the Golgi-to-plasma membrane transport of GPCRs

**Timing: 4 days**

To study the Golgi-to-plasma membrane transport of GPCRs, the cells are transfected with GPCR RUSH plasmids for 20 h and incubated with biotin at 20°C for 3 h. This temperature allows nascent receptors to export from the ER to the Golgi, but the receptors are unable to export from the Golgi. After 3 h biotin induction, all nascent receptors will be accumulated in the Golgi and their transport from the Golgi will be synchronized after incubating the cells at 37°C (Figure 1).

34. Transfect the cells with 500 ng of Str-KDEL_SBP-EGFP-GPCR for 20 h by using PEI solution following steps 11–15.
35. Add ice to water bath and set the temperature at 20°C. Incubate DMEM containing FBS, biotin and CHX at 20°C in water bath for at least 20 min.
36. Incubate the cells at 20°C in water bath for 10 min. Remove the medium and add 1 mL of DMEM with FBS, biotin and CHX. Keep the cells at 20°C for 3 h.
37. Incubate DMEM containing FBS and CHX at 37°C for 10 min.
38. For control well, wash the cells twice with 1 mL of cold PBS and fix the cells with 4% PFA solution following steps 17–18.
39. To initiate post-Golgi transport, add 1 mL of pre-warmed DMEM with FBS and CHX in each well. To study the transport kinetics, the cells are incubated at 37°C for different time periods (for example, 15, 30, 45 and 60 min).

***Note:*** It is recommended to use different cell culture plates for different time points.

40. Wash the cells twice with 1 mL of PBS, fix the cells and mount the coverslips following steps 17–18 .
41. Image acquisition following steps 22–23.
42. Measure receptor expression in the Golgi and the whole cell before (0 min) and after incubation at 37°C and calculate the Golgi/total expression ratios of the receptor following step 33.

**Figure 5:**
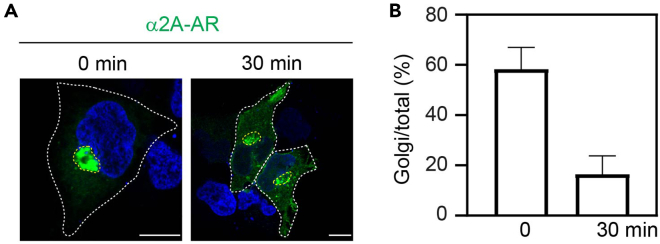
Analysis of the post-Golgi transport of α_2A_-AR (A) Post-Golgi transport of α_2A_-AR in HEK293 cells. The cells were transfected with Str-KDEL_SBP-EGFP-α_2A_-AR for 20 h, then treated with biotin and CHX at 20°C for 3 h (0 min) and incubated with fresh DMEM without biotin at 37°C for 30 min (30 min). (B) Quantification of the Golgi/total expression ratios of α_2A_-AR before and after incubation at 37°C for 30 min following biotin induction at 20°C for 3 h. The quantitative data are expressed as mean ± SD (n = 25-43 cells from 3 experiments). Scale bars, 10 μm.

***Note:*** α_2A_-AR is used as an example to analyze the post-Golgi transport (Figure 5A). The Golgi/total expression ratio of α_2A_-AR at 0 min is about 60% and this ratio decreases to less than 20% after incubation for 30 min at 37°C (Figure 5B).

## Expected outcomes

By analyzing the colocalization of individual GPCRs with the COPII component Sec24D before biotin addition in RUSH assays, we expect that certain GPCRs are able to colocalize with Sec24D in the punctate structures in the cell periphery. If so, these receptors are concentrated at the ERES and actively captured by COPII vesicles (Figures 3A–3C). For most GPCRs, they are evenly distributed throughout the ER before biotin incubation, suggesting that these receptors are not concentrated in COPII vesicles.

Biotin incubation synchronizes ER export of nascent GPCRs. After measuring the Golgi/total expression ratios at different time points after biotin induction, the ER-to-Golgi transport kinetics can be defined. It is expected that distinct GPCRs may have different transport kinetics along the early secretory pathway.

To study the post-Golgi transport of GPCRs, the RUSH system is combined with low temperature culture of cells which blocks the receptors to export from the Golgi. Similar to the ER-to-Golgi transport, Golgi-to-cell surface transport can be quantified after releasing the receptors from the Golgi for different time. It is also expected that different GPCR members may have different post-Golgi transport kinetics.

## Limitations

One limitation of the protocol described here to study the COPII concentration and forward delivery of nascent GPCRs is that GPCR expression via the RUSH system is extremely high as compared to their expression at the endogenous levels. As such, COPII concentration and export kinetics of individual GPCRs as observed in the RUSH system may not reflect the transport properties of endogenous receptors.

Another potential issue is that in the RUSH system, GPCR of interest is conjugated with a fluorescent protein and SBP which may affect the correct folding and proper assembly of the receptor, thus disrupting receptor recruitment to COPII vesicles and/or export from the ER. Therefore, the receptor to be studied in the RUSH system should be carefully characterized as described in the section “characterization of RUSH plasmids of GPCRs”.

## Troubleshooting

### Problem 1

GPCRs remain in the ER, unable to response to biotin induction (related to steps 16–23).

### Potential solution

The position of fluorescent tag may affect GPCR correct folding and misfolded receptors are unable to pass through the ER equality control and retained in the ER. One approach to tackle this issue is to generate GPCR RUSH plasmids with the fluorescent tag at different termini and test different fluorescent tags, such as GFP, mCherry and DsRed.

### Problem 2

The KDEL hook is unable to arrest nascent GPCRs in the ER (related to steps 16–23).

### Potential solution

The KDEL sequence has been used in our studies as an ER hook and worked quite well to block ER export of a number of GPCRs.^1^^,^^2^^,^^22^ If GPCR of interest is able to transport out of the ER in the absence of biotin, other ER hooks, such as a mutant of stromal interaction molecule 1 (STIM1-NN),^21^ can be tested.

### Problem 3

GPCRs escape from the ER before treatment with biotin (related to steps 16–23).

### Potential solution

If a significant portion of nascent GPCRs leak from the ER before adding biotin to cell culture medium to induce receptor export, one potential cause is the presence of biotin in FBS. Biotin-depleted FBS can be used to reduce the leakage. Alternatively, Dynabeads MyOne streptavidin beads (Thermo Fisher Scientific) can be used to remove biotin from FBS.

### Problem 4

GPCRs are visualized to be clearly concentrated in vesicles under the microscope, but the vesicles cannot be captured due to quenching (related to steps 26–27).

### Potential solution

Once the cells containing COPII vesicles are identified, turn off the light and adjust the intensity and laser power quickly. Capture the image at a lower speed (400 or 600). The LIGHTNING model can be used to enhance the imaging resolution.

### Problem 5

In post-Golgi transport experiments, cells die after long incubation out of incubator at low temperature (related to step 36).

### Potential solution

If available, refrigerated incubator can be used for low temperature culture. In addition, the Golgi hooks, such as Golgin-84,^21^ can be tested to anchor the receptors in the Golgi which can be released after biotin incubation.

## Resource availability

### Lead contact

Further information and requests for resources and reagents should be directed to and will be fulfilled by the lead contact, Guangyu Wu (guwu@augusta.edu).

### Technical contact

Please direct technical questions regarding this protocol to the technical contact, Xin Xu (xinxu@augusta.edu).

### Materials availability

Reagents generated in this study are available from the lead contact upon request.

### Data and code availability

• All data reported in this paper will be available from the lead contact upon request.
• This paper does not report the original code.
• Any additional information reported in this paper will be shared by the lead contact upon request.
